# STELLA Facilitates Differentiation of Germ Cell and Endodermal Lineages of Human Embryonic Stem Cells

**DOI:** 10.1371/journal.pone.0056893

**Published:** 2013-02-14

**Authors:** Patompon Wongtrakoongate, Mark Jones, Paul J. Gokhale, Peter W. Andrews

**Affiliations:** Centre for Stem Cell Biology, Department of Biomedical Science, University of Sheffield, Sheffield, United Kingdom; University of Melbourne, Australia

## Abstract

*Stella* is a developmentally regulated gene highly expressed in mouse embryonic stem (ES) cells and in primordial germ cells (PGCs). In human, the gene encoding the STELLA homologue lies on chromosome 12p, which is frequently amplified in long-term cultured human ES cells. However, the role played by STELLA in human ES cells has not been reported. In the present study, we show that during retinoic acid (RA)-induced differentiation of human ES cells, expression of *STELLA* follows that of *VASA*, a marker of germline differentiation. By contrast, human embryonal carcinoma cells express *STELLA* at a higher level compared with both karyotypically normal and abnormal human ES cell lines. We found that over-expression of STELLA does not interfere with maintenance of the stem cell state of human ES cells, but following retinoic acid induction it leads to up-regulation of germline- and endodermal-associated genes, whereas neural markers *PAX6* and *NEUROD1* are down-regulated. Further, STELLA over-expression facilitates the differentiation of human ES cells into BE12-positive cells, in which the expression of germline- and endodermal-associated genes is enriched, and suppresses differentiation of the neural lineage. Taken together, this finding suggests a role for STELLA in facilitating germline and endodermal differentiation of human ES cells.

## Introduction

*Stella/Dppa3/PGC7* was originally identified in mouse pre-implantation embryos, PGCs and developing germ cells, where it localizes in both nucleus and cytoplasm [1], [2]. The protein is very basic with an isoelectric point of about 9 and a molecular weight of about 17 kilodalton. It has been proposed to carry both putative nuclear import and export signaling domains, a SAP-like domain and a splicing factor motif-like domain, suggesting that the protein might participate in regulation of chromatin and/or RNA binding [1], [2]. Surani and colleagues have shown that, in the mouse, *Stella* expression is repressed in the post-implantation epiblast, and re-established again at a high level in PGCs at E7.25, and that the expression of homeobox genes, *Hoxa1* and *Hoxb1,* is down-regulated in *Stella*-positive PGCs compared with *Stella*-negative cells [2], [3]. This finding implies that PGCs have undergone the germ cell specification program with a similar timing to the repression of somatic cell program. However, in mouse embryos, although emerging PGCs of the post-implantation embryo up-regulate Stella expression, it is not required for PGC specification. In addition, *Stella-*null pups are viable. Nevertheless, *Stella*-null female mice show a reduction in fertility because Stella is required as a maternal inheritance factor in oocytes [3], [4]. The role of Stella in maternal effect inheritance is supported by its high expression in oocytes [1]. Interestingly, Nakano and colleagues have found that maternally inherited Stella in the zygote prevents global, active DNA demethylation of the maternal genome, while the paternal genome undergoes genome-wide active DNA demethylation emphasizing the function of *Stella* in survival of embryos by safe-guarding the maternal genome, particularly at genomic imprinted loci [5].

The role of Stella beyond the zygotic stage of developing mouse embryos is poorly understood. Until recently, *Stella* expression status has been linked to choices of differentiation of mouse embryonic stem (ES) cells [6]. *Stella*-positive and -negative mouse ES cells are interchangeable reflecting the naïve and primed states of mouse pluripotent stem cells, respectively. Specifically, *Stella*-negative mouse ES cells show a gene expression profile similar to that of mouse epiblast stem cells. In addition, *Stella*-negative ES cells acquire a higher propensity for neural and trophectoderm differentiation compared with *Stella* ^high^ ES cells. This differentiation bias is supported by an up-regulation of *Pax6* and *Cdx2*, which are the master regulators of neural and trophoblast differentiation, respectively, in *Stella*-negative ES cells. Hence, Stella might possess a significant functional role in suppressing differentiation of mouse ES cells toward neuroectodermal and trophectodermal lineages [6].

The human *STELLA* gene homologue is located on chromosome 12p13, and lies between *GDF3* and *NANOG*, both which are highly expressed in ES and embryonal carcinoma (EC) cells [7], [8], [9]. However, in contrast to mouse ES cells in which the *Stella* promoter is demethylated allowing a high expression level of the gene to be transcribed, the *STELLA* promoter in human ES cells has been shown to be methylated, like that of mouse epiblast stem cells [6], [10]. Similar to PGC specification of mouse embryos, *STELLA* is up-regulated during a directed germ cell differentiation of human ES cells [11], and is co-expressed with *VASA*, a definitive germ cell marker, in germ cell differentiating conditions [12]. Therefore, *STELLA* up-regulation in differentiating human ES cells might indicate the appearance of an *in vitro* counterpart of human PGCs. Interestingly, the genomic region of 12p13 is frequently duplicated in long-termed culture of human ES cells [13], [14], and also in human EC and seminoma cells [15], [16]. Although EC and seminoma cells share many characteristics, studies of gene expression profile between the two types of testicular germ cell tumor have shown distinguishing features. Specifically, high expression of *STELLA* and *SOX2* is observed in EC cells compared with seminoma and vice versa for *SOX17* [8], [17], [18], [19]. In fact, *SOX17* has been proposed as a useful marker to distinguish seminoma from EC cells [17] and is down-regulated in differentiated seminoma cell line TCam-2 [20], [21]. Since the chromosomal gain of this region is a hallmark of EC and seminoma cells, amplification of this region might therefore provide a selective advantage to the so-called culture adapted human ES cells. Whether STELLA gain-of-function plays an important role in survival of culture adapted human ES cells has not been described.

In this study, we report the role of *STELLA* in facilitating early germ cell and endodermal differentiation of human ES cells. We found that over-expression of STELLA does not promote maintenance of the stem cell state of human ES cells. On the other hand, similar to mouse ES cells, STELLA over-expression suppresses expression of trophectodermal- and neural-associated genes, while germline- and endodermal-associated genes are up-regulated during induced differentiation. Further, *STELLA* over-expression facilitates the generation of cells expressing the surface antigen, BE12 [22], which might represent cells of an early germ cell developmental stage and endodermal lineage. These results support a role for *STELLA* in supporting the germ cell and endoderm differentiation programmes of human ES cells.

## Results

**Figure 1:**
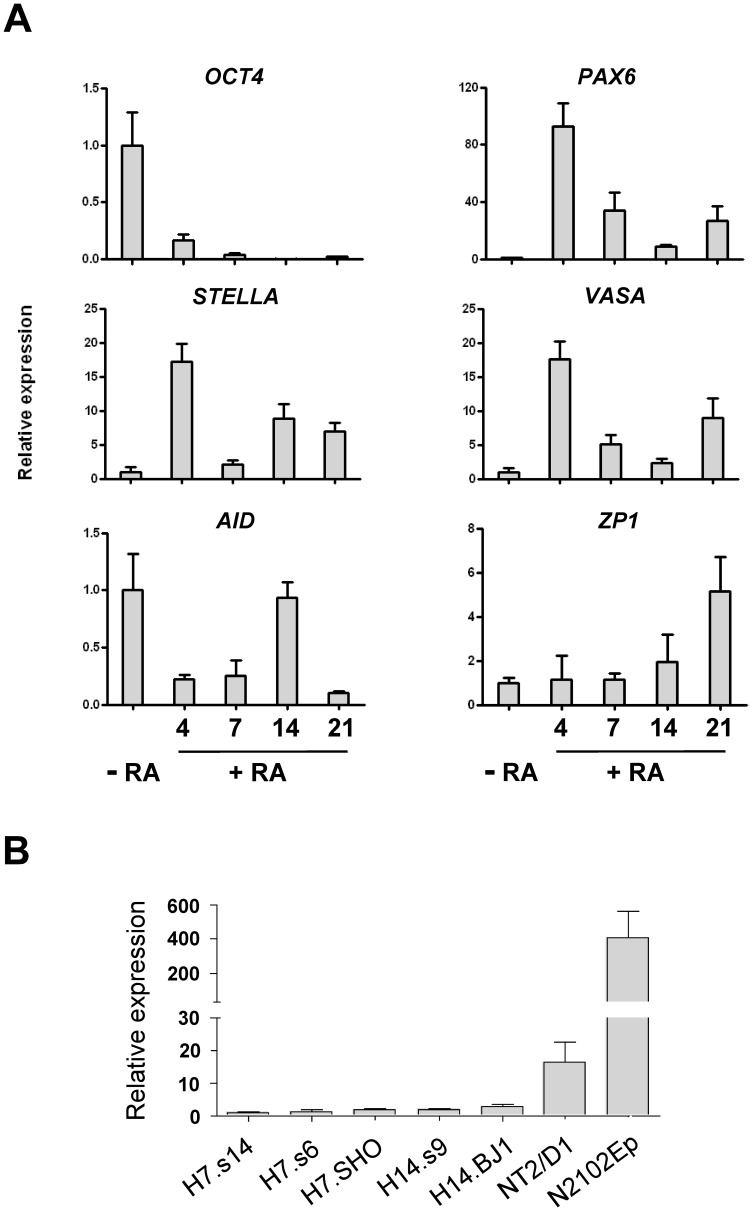
Expression of *STELLA* during the differentiation of human ES cells. (A) *STELLA* expression is up-regulated during RA-induced differentiation of human ES cells. RT-qPCR analysis was performed for genes specifically expressed in undifferentiated stem cells (*OCT4*), neural differentiation (*PAX6*) and germ cells (*STELLA*, *VASA*, *AID* and *ZP1*) during RA-induced differentiation of human ES cells. (B) Human EC cells highly express *STELLA* compared with human ES cells. RT-qPCR was used to test *STELLA* expression in normal and karyotypically abnormal sublines of human ES celllines, H7.s14 [46,XX], H7.s6 [47,XX, +1, der(6)t(6;17)(q27;q1)], H7.SHO [48,XX,+1,der(6)t(6;17)(q27;q1),+8], H14.s9 [46,XY] and H14.BJ1 [47,XY, +12], and in human EC cell lines NT2/D1 and N2102Ep. Cytogenetic analysis of the latter two cell lines has been described elsewhere [9]. Data are shown as mean±SD; n  =  3.

We first investigated the expression profile of *STELLA* in human ES cells and during their differentiation induced by all-*trans*-retinoic acid (RA). The expression of *STELLA* was low in undifferentiated human ES cells, but it was up-regulated, together with *VASA* and *ZP1*, during differentiation, while *OCT4* was repressed (Figure 1A). *AID*; a gene that has been reported to be highly expressed in both ES cells and PGCs [23], was down-regulated during day 4 and day 7 of differentiation. Its expression was then briefly up-regulated again on day 14. Expression of *PAX6*, a neural commitment gene known to be induced by RA, was also activated in RA-induced differentiation, consistent with somatic differentiation towards neural cells. Since the gene encoding STELLA is located on chromosome, in the region 12p13, which is frequently subject to amplification during culture adapted human ES cells, and in EC cells [13], [14], we also assessed expression *STELLA* in karyotypically normal versus abnormal human ES cells together with human EC cells N2102Ep and NTERA2 (NT2/D1). We found that human EC cells express *STELLA* at a significantly higher level compared with human ES cells, in which expression was low, whether or not they carried karyotypic abnormalities including gain of chromosome 12 (Figure 1B). This difference in *STELLA* expression might reflect the origin of EC cells from PGC.

**Figure 2:**
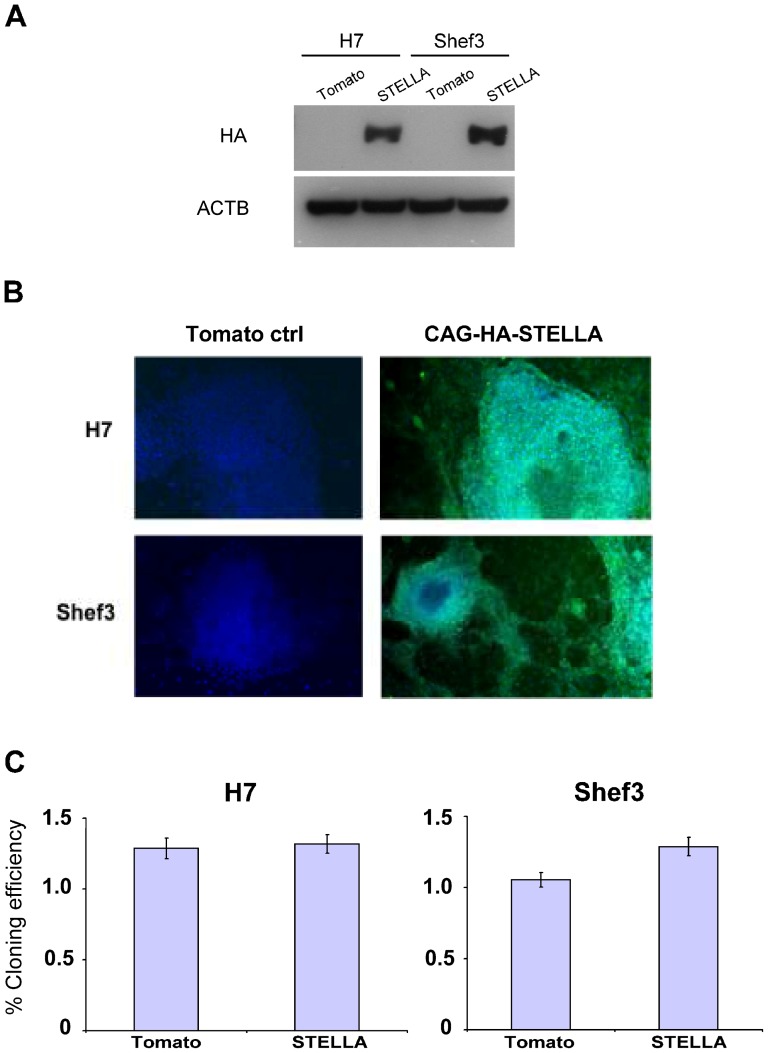
Establishment of STELLA over-expression cell lines from human ES cells. Clones of human ES cells over expressing the fluorescent protein ‘Tomato’ or HA-tagged STELLA were isolated after transfection with vectors encoding Tomato or HA-STELLA under the control of the CAG constitutive promoter. (A) Western blot analysis of HA tag in human ES cells H7 and Shef3 over-expressing Tomato or HA-tagged STELLA. (B) Immunostaining of ectopically expressed STELLA using HA tag antibody. (C) STELLA over-expression does not influence clonal ability of human ES cells. Colony number was counted and reported as percent cloning efficiency. Data are shown as mean±SD; n  =  3.

To ascertain whether *STELLA* expression influences the undifferentiated and/or differentiated state of human ES cells, we transfected two human ES cell lines, H7 (46,XX) and Shef3 (46,XY), with a plasmid containing STELLA coding sequence with HA tag at its N-terminus driven by CAG promoter [24] to establish human ES cells over-expressing STELLA. Control cell lines of H7 and Shef3 were established using a CAG plasmid over-expressing Tomato fluorescent protein as to assess the transfection and selection of stable cell lines by puromycin. Western blot analysis using anti-HA antibody showed that the human ES cell lines transfected with the STELLA-HA-tag vector did indeed over-express STELLA, whereas control lines transfected to over-express the Tomato fluorescent protein do not express the transgene (Figure 2A). Immunostaining of HA tag also revealed an over-expression of exogenous STELLA in ES cells (Figure 2B).

**Figure 3:**
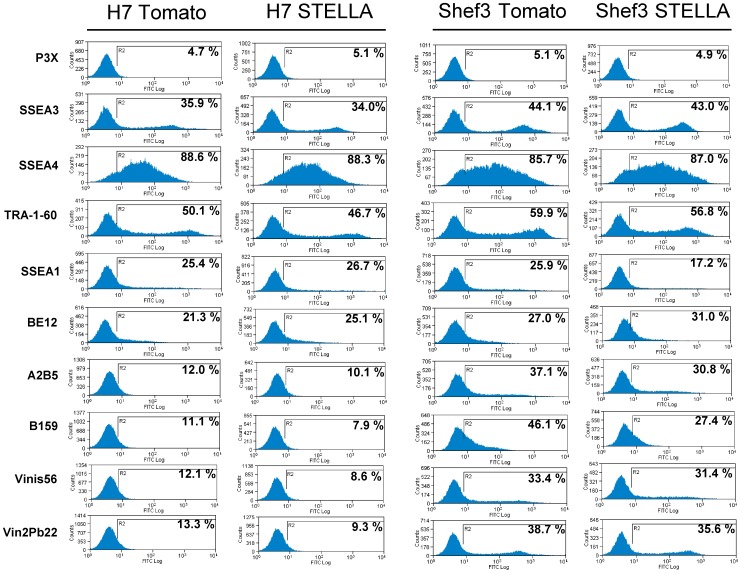
Expression of cell surface markers in human ES cells over-expressing STELLA. Flow cytometric analysis of various surface antigens characteristic of human ES cells or their differentiated derivatives in human ES cells, H7 and Shef3, transfected with Tomato or with STELLA. The expression of these marker antigens was not affected by STELLA over-expression.

Clongenicity assays were then performed to determine whether STELLA influences the stem cell state of human ES cells. However, over-expression of STELLA did not affect cloning efficiency of human ES cells (Figure 2C). Consistent with this, the pattern of expression of surface antigens characteristic of the undifferentiated cells, or their differentiated derivatives, was unaffected by over expression of STELLA in cultures maintained under stem cell conditions (Figure 3).

**Figure 4:**
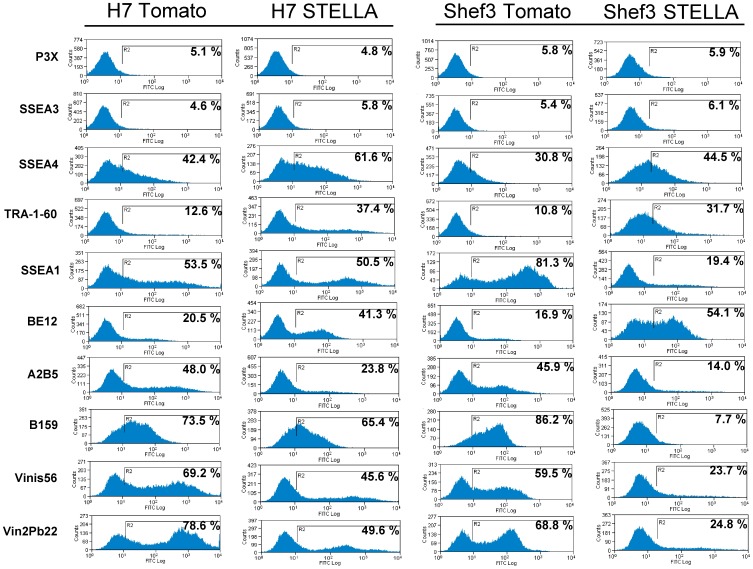
Expression of cell surface markers during RA-induced differentiation of human ES cells over-expressing STELLA. Human ES cell lines H7 and Shef3 over-expressing Tomato or STELLA were induced to differentiate by 10 µM RA for 7 days. Expression of surface markers was determined by flow cytometry. STELLA over-expression facilitates generation of BE12-positive cells and tends to inhibit generation of differentiated cells expressing antigens characteristic of neural differentiation (A2B5, VINIS56 and VIN2PB22, recognnising gangliosides GT3, GD3 and GD2, and B159, recognizing NCAM) (.

By contrast, over expression of STELLA did affect the pattern of differentiation observed after RA-induction. In the control cells, expressing Tomato, the expression of stem cell markers SSEA-3, SSEA-4 and TRA-1-60 declined 7 days after initial exposure to 10 µM RA, whereas the expression of cell surface antigens characteristic of neural differentiation, A2B5 (ganglioside GT3), B159, VINIS56 (ganglioside GD3), VIN2Pb22 (ganglioside GD2), and B159 (NCAM) was induced (Figure 4) [25]. However, the STELLA over-expressing cells showed a higher number of cells expressing SSEA-4 and TRA-1-60, and also gave rise to more cells expressing the novel surface antigen, BE12,compared with the control cell lines (Figure 4). On the other hand, the STELLA over-expressing ES cells produced fewer cells expressing the markers of neural differentiation, A2B5, VINIS56, VIN2Pb22, and B159. This suggested that expression of STELLA altered the lineage bias of the ES cells away from neural differentiation towards lineages marked by the surface marker BE12.

**Figure 5:**
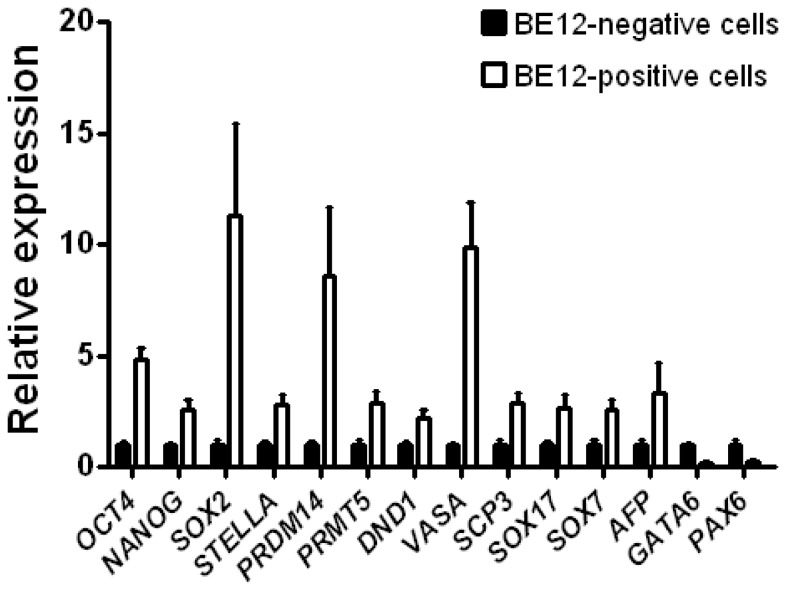
Gene expression of BE12-positive cells. Human ES cells were induced to differentiate by 10 µM RA for 7 days. Differentiated cells were trypsinized and stained for BE12. BE12 positive and negative cells were isolated by Fluorscence Activarted Cell Sorting, and were subjected to RT-qPCR analysis. Expression of genes associated with germ cell and endodermal lineages was enriched in BE12-positive cells. Data are shown as the mean±SD; n  =  3.

BE12 is a novel monoclonal antibody produced by a hybridoma that was generated after using human ES cells for immunization, but that reacts more strongly with human EC and endodermal cell lines [22]. To characterise the developmental lineage(s) marked by BE12, and to test whether these might represent germ cell and/or endodermal lineages, we analysed the expression of several genes in cells sorted for BE12 expression from day 7 RA-induced human ES cultures. The BE12-positive cells expressed *STELLA*, *PRDM14*, *PRMT5*, *DND1*, *VASA*, *SCP3*, *SOX17*, *SOX7* and *AFP*, genes found in both germ cell and endodermal lineages [23], [26], [27], [28], [29], [30], at a higher level than BE12-negative cells (Figure 5). By contrast, the expression of *GATA6* and *PAX6*, which are the master regulators of extraembryonic endodermal and neural lineages, respectively, was lower in the BE12-positive cells than in the BE12-negative cells. These results suggest that the BE12-positive cells induced by *STELLA* over-expression may represent both early germline and definitive endodermal cells.

**Figure 6:**
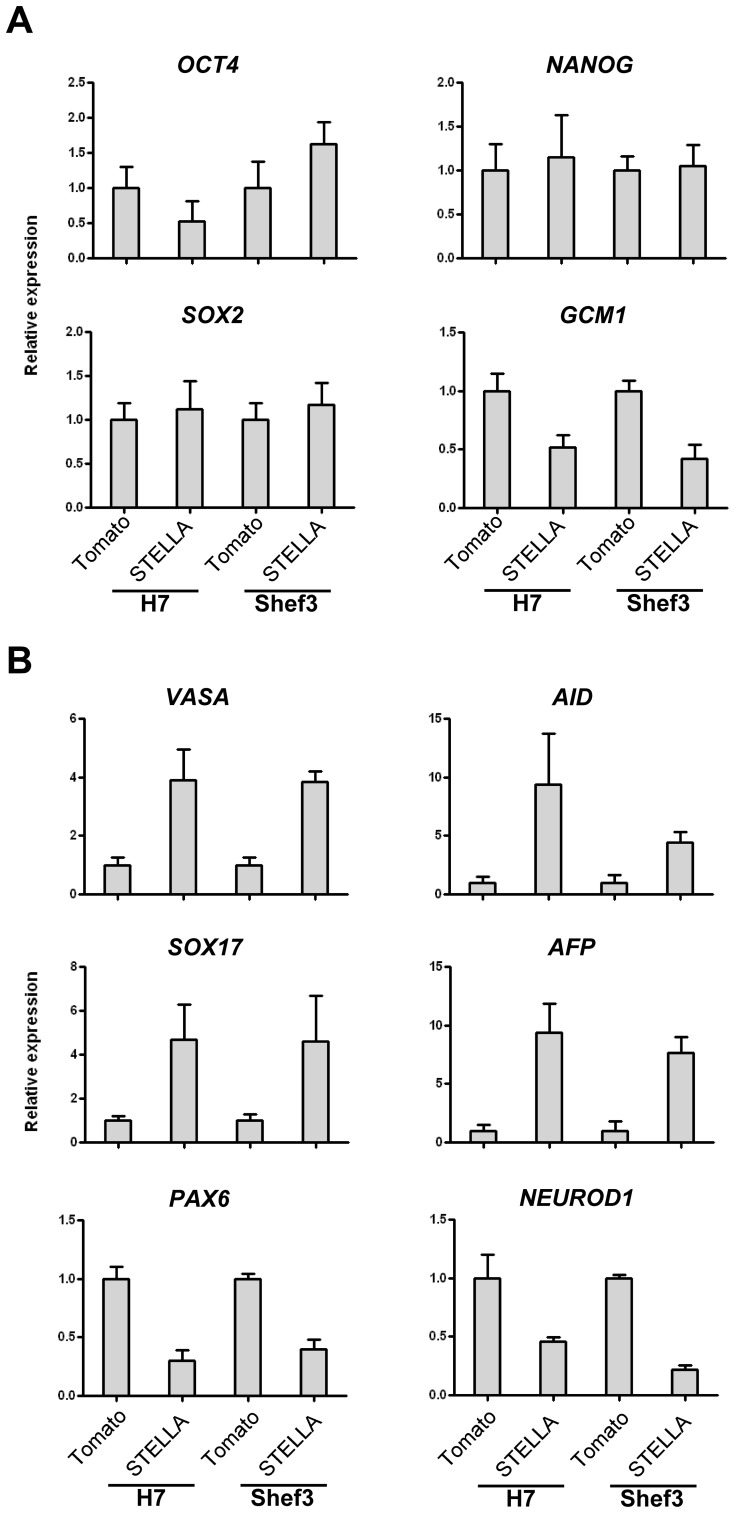
Differential gene expression of human ES cells expressing STELLA. (A) RT-qPCR analysis of human ES cells over-expressing STELLA grown under undifferentiated condition. STELLA over-expression suppresses expression of a trophectodermal gene *GCM1*. (B) RT-qPCR analysis of RA-induced human ES cells over-expressing STELLA. STELLA induces genes involved in germ cell development *VASA* and *AID*, but suppresses expression of neural differentiation genes *PAX6* and *NEUROD1*. Data are shown as mean±SD; n  =  3.

Consistent with the assays for clonogenicity and antigen expression, the over expression of STELLA in the undifferentiated culture of human ES cells did not affect the expression of stem cell-associated genes *OCT4* and *NANOG* (Figure 6A), although the trophectoderm associated gene, *GCM1*, was down-regulated suggesting that STELLA might inhibit spontaneous trophoectodermal differentiation in these cultures. On the other hand, genes expressed in germline and endodermal cells, including *VASA*, *AID*, *SOX17* and *AFP*, were up-regulated after RA-induced differentiation, whereas neural markers *PAX6* and *NEUROD1* were down-regulated in cells over-expressing STELLA, in comparison to control cells (Figure 6B). Collectively, these results indicate that STELLA does not promote maintenance of stem cell state of human ES cells, but rather influences fate determination towards germ cell and endodermal lineages upon induction of differentiation.

## Discussion

In this study, we found that the pattern of *STELLA* expression during RA-induced differentiation of human ES cells follows that of *VASA* expression and other genes associated with the germ line (Figure 1A). This gene expression pattern supports the notion of germline differentiation of human ES cells following RA induction [12], and suggests that the activation of *STELLA* is an early event of germ cell differentiation of human ES cells.

Although *Stella* is highly expressed in mouse ES cells [6], [8], and in human EC cells (Figure 1B), the over-expression of STELLA in human ES cells does not promote clongenicity of human ES cells (Figure 2C and 3). Gain of the short arm of chromosome 12, which harbors *STELLA* as well as the stem cell-associated gene *NANOG*, is common in both human EC cells and in long-term cultured human ES cells [13], [14]. However, our results suggest the selective advantage thought to associated with gain of chromosome 12 is not dependent upon the over-expression of STELLA. On the other hand, STELLA over-expression results in more cells expressing the surface antigen, BE12, and less cells with neural markers in RA-induced human ES cell differentiation than the control (Figure 4). The BE12 cell surface antigen is particularly strongly expressed by some human EC and yolk sac carcinoma cell lines but only weakly and sporadically by human ES cells [22]. In the current study, we found that BE12-positive human ES cells may represent early germline and definitive endoderm differentiation. The expression of BE12 in human EC cells might reflect their origin from primordial germ cells, and perhaps less than complete reversion to an embryonic state resembling that of human ES cells. Further characterization of BE12-positive cells derived from human ES culture in more detail might provide a new tool to study germline and endodermal differentiation of human ES cells.

That over-expression of STELLA leads to the repression of genes and surface antigens associated with trophectodermal and neural differentiation, but enhances the the expression of genes associated with definitive endoderm and the germline suggests a role for STELLA in lineage selection of human ES cells. Our results are therefore consistent with the finding from Surani and colleagues, which shows that mouse ES cells with a higher level of *Stella* expression exhibit a lower expression level of trophectodermal and neural markers than those with a lower level of *Stella* expression [6].

A strong up-regulation of *Stella* has been observed in mouse PGCs after specification from post-implantation epiblast [2]. Although not required for PGC differentiation and survival in mice [3], [4], its function in the germline might be to directly promote expression of germ cell-associated genes and/or to suppress trophectodermal and somatic programs through its DNA binding domain [31]. Another possible function of Stella in the germline might be to prevent active DNA demethylation at loci other than imprinted genes during the erasure of genomic imprinting status in PGCs [32], since it protects maternal genome from DNA demethylation in the zygote [5]. The increase in *AID* expression of differentiated human ES cells over-expressing STELLA might therefore mimic the PGC reprogramming event, because AID is implicated in active DNA demethylation process [33], [34]. Since human ES cells have been proposed to be similar to mouse epiblast stem cells [35], [36], it would be of interest to test whether over-expression of Stella in mouse epiblast stem cells will result in germline and/or endodermal differentiation. In fact, up-regulation of Stella in mouse epiblast stem cells during germline differentiation has been described elsewhere [29]. Furthermore, DAZL and BOULE, which are highly expressed in the germline, have been shown to promote *in vitro* generation of PGCs from human ES cells [12]. Therefore, an over-expression of STELLA in human pluripotent stem cells together with those germline inducing genes might synergistically facilitate differentiation of the stem cells toward germ cell lineage.

## Materials and Methods

### Cell culture

Human ES cell lines H7 and Shef3 [7], [37] were grown in Knockout-DMEM supplemented with 20% Knockout-Serum Replacement, 1X non-essential amino acids, 1 mM glutamine, 0.1 mM beta-mercaptoethanol and 4 ng/ml bFGF (Invitrogen) seeded on 6×10^3^ cells/cm^2^ mitomycin C-treated MF-1 mouse embryonic fibroblasts (MEFs), and cultured at 37°C under a humidified atmosphere of 5% CO_2_ in air. Cells were passaged every 5–7 days using collagenase type IV (Invitrogen) and scraping with glass-beads (Sigma). To induce differentiation of human ES cells, the culture medium was supplemented with 10 µM of all-*trans*-retinoic acid (Sigma).

### Construction of human ES cells over-expressing *STELLA*

cDNA encoding the human STELLA homologue was amplified using One-Step RT-PCR kit (Invitrogen). A haemagglutinin (HA) sequence tag-containing primer was used to add the HA tag at the 5’-position of the cDNA to allow detection of the transgene by staining the HA tag. The HA-STELLA DNA fragment was gel-purified using a DNA extraction kit from Fermentas. The Tomato encoding gene of pCAG-Tomato-IRES-puromycin [38] was excised using *XhoI* and *NotI*, and was replaced by the HA-STELLA DNA fragment. The plasmid pCAG-HA-STELLA-IRES-puromycin was then transfected into either H7 or Shef3 to establish the STELLA over-expressing cell lines using electroporation. Briefly, human ES cells were dissociated using 0.05 % trypsin. One million human ES cells were then transfected with 5 µg *PmlI*-linearized plasmid DNA. pCAG-Tomato-IRES-puromycin was also separately transfected in the parental H7 and Shef3 to construct control cell lines. The transfectants were immediately plated on MEFs with human ES culture medium. One week after the transfection, 1 µg/ml puromycin was added to the culture medium to select human ES cell colonies which were resistant to the antibiotic. RT-qPCR and immunofluorescence staining were employed to test STELLA over-expression in the human ES cell lines. STELLA over-expressing clones of human ES cell lines H7 and Shef3 that show the highest expression level of STELLA transgene were investigated in further experiments.

### Reverse transcription quantitative polymerase chain reaction (RT-qPCR)

RNA was extracted using TRIzol reagent (Invitrogen). Complementary DNA synthesis was performed with 1 µg RNA. qPCR was carried on by using SYBR Green JumpStart *Taq* ReadyMix (Sigma) in a total volume of 20 µl each well with an iCycler iQ system (Biorad). Gene expression was normalized by expression level of *ACTB*. Primer sequences are available upon request.

### Immunofluorescence staining

Cells were grown in a 24-well chamber until nearly confluent, and were fixed with 4% PFA. Fixed cells were permeabilized with 0.5% Triton-X 100, and were incubated with an antibody against rabbit anti-HA tag (Abcam). A secondary antibody conjugated with FITC (Santa Cruz) was then used for visualization under an InCell Analyzer fluorescence microscope workstation system (GE Healthcare).

### Flow cytometry and cell sorting

Flow cytometry was performed as previously described [22], [25], [39]. Briefly, cultured cells were harvested by trypsinization. One hundred thousand cells in 100 µl PBS with 10% FCS were incubated with primary monoclonal antibodies including P3X (negative control), SSEA-3, SSEA-4, TRA-1-60, SSEA-1, BE12, A2B5, B159, Vinis56 and Vin2Pb22 at 1:10 dilution for 30 min [25]. Cells were washed and incubated with FITC-conjugated secondary antibody (Jackson Laboratory). Cell surface expression was analyzed by a Cyan bench-top flow cytometer (Dako Cytomation). To isolate BE12-positive and –negative populations 10^7^ human ES cells were incubated with BE12 antibody at 1 10 dilution in 10 ml PBS with 10% FCS. The cells were washed, incubated with the secondary antibody for 30 min, and were sorted by a Moflo flow cytometer system (Dako Cytomation) into 1 ml PBS with 10% FCS. The sorted cells of both negative and positive BE12 populations were extracted for RNA using Trizol (Invitrogen).
